# UTILIZATION TRENDS OF GLUCOSE-LOWERING DRUGS FOR TYPE 2 DIABETES IN OLDER ADULTS WITH AND WITHOUT DEMENTIA

**DOI:** 10.1093/geroni/igac059.1819

**Published:** 2022-12-20

**Authors:** Chandrasekar Gopalakrishnan, Dae Kim, Elisabetta Patorno

**Affiliations:** Brigham and Women's Hosp./Harvard Medical School, Boston, Massachusetts, United States; Hebrew SeniorLife, Boston, Massachusetts, United States; Brigham and Women's Hospital, Harvard Medical School, Boston, Massachusetts, United States

## Abstract

Dementia is an important consideration in the therapeutic management of older adults with type 2 diabetes (T2D). Given the recent availability of numerous second-line therapeutic agents, there is limited evidence on uptake of these newer medications by dementia status. Using Medicare fee-for-service data from 2013-18, we identified a cohort of patients with T2D who initiated a glucose-lowering drug (N=3,355,725; mean [SD] age, 74.8 (6.9) years) and stratified our analysis based on the presence of a diagnosis for dementia in the year prior to treatment initiation. Amongst patients with dementia (N=511,835; mean [SD] age, 79.9 (7.7) years), metformin use remained stable from 25.8% to 24.8%, whereas sulfonylureas (20% to 17.5%) and insulin (31.8% to 26.2%) use declined. Amongst patients without dementia (N=2,843,890; mean [SD] age, 73.8 (6.3) years), metformin (31.7% to 24.7%), sulfonylurea (22.1% to 19.4%) and insulin use (18.7% to 14.6%) decreased. DPP-4i and glitazones use remained largely stable whereas the use of newer agents such as SGLT-2i and GLP-1 RA increased steadily in both patients with and without dementia though the uptake was much higher in patients without dementia. By the end of 2018, 19.6% of patients initiated either a SGLT-2i or a GLP-1 RA amongst those without dementia whereas only 11.1% did so amongst those with dementia. In conclusion, older medications such as metformin, sulfonylureas and insulin accounted for about two-thirds of initiated glucose-lowering medications and were more frequently used by patients with dementia, though their use declined steadily over time with the availability of newer agents.

